# Post-transcriptional Regulation of PCSK9 by miR-191, miR-222, and miR-224

**DOI:** 10.3389/fgene.2017.00189

**Published:** 2017-11-27

**Authors:** Parisa Naeli, Fatemeh Mirzadeh Azad, Mahshid Malakootian, Nabil G. Seidah, Seyed J. Mowla

**Affiliations:** ^1^Department of Molecular Genetics, Faculty of Biological Sciences, Tarbiat Modares University, Tehran, Iran; ^2^Cardiogenetic Research Center, Rajaie Cardiovascular Medical and Research Center, Iran University of Medical Sciences, Tehran, Iran; ^3^Laboratory of Biochemical Neuroendocrinology, Clinical Research Institute of Montreal, Montreal, QC, Canada

**Keywords:** microRNA, PCSK9, miR-191, miR-222, miR-224

## Abstract

Since proprotein convertase subtilisin kexin 9 (PCSK9) discovery, a gene involved in LDL metabolism regulation and cardiovascular diseases (CVD), many therapeutic strategies have been introduced for direct targeting of PCSK9. The main goal of these strategies has been to reduce PCSK9 protein level either by application of antibodies or inhibition of its production. In this study, we have tried to discover microRNAs (miRNAs) which can target, and hence regulate, PCSK9 expression. Using bioinformatics tools, we selected three microRNAs with binding sites on 3′-UTR of PCSK9. The expression level of these miRNAs was examined in three different cell lines using real-time RT-PCR. We observed a reciprocal expression pattern between expression level of miR-191, miR-222, and miR-224 with that of PCSK9. Accordingly, the expression levels were highest in Huh7 cells which expressed the lowest level of PCSK9, compared to HepG2 and A549 cell lines. PCSK9 mRNA level also showed a significant decline in HepG2 cells transfected with the vectors overexpressing the aforementioned miRNAs. Furthermore, the miRNAs target sites were cloned in psiCHECK-2 vector, and a direct interaction of the miRNAs and the PCSK9 3′-UTR putative target sites was investigated by means of luciferase assay. Our findings revealed that miR-191, miR-222, and miR-224 can directly interact with PCSK9 3′-UTR and regulate its expression. In conclusion, our data introduces a role for miRNAs to regulate PCSK9 expression.

## Introduction

An estimated 17.5 million people died from cardiovascular diseases (CVDs) in 2012, representing ~31% of all global deaths. Of these death numbers, roughly 7.4 million were due to coronary heart disease and 6.7 million were due to stroke (WHO, 2016). Hypercholesterolemia, one of the major risk factors for atherosclerosis and developing CVD, is characterized by high level of low density lipoprotein (LDL) cholesterol (LDL-C) in plasma (Newman et al., 1986; Lusis, 2000; Hopkins et al., 2011; Varghese, 2014; Bruikman et al., 2017). LDL particles are removed from the circulation mainly by hepatic uptake via the LDL receptor (LDLR). LDL binds to the LDLR and the LDL/LDLR complex is internalized via clathrin-coated vesicles. Then, LDL is separated from its receptor in the endosomes and the LDLR is recycled back to cell surface, while LDL is degraded (Kwiterovich, 2000; Goldstein and Brown, 2009).

PCSK9 has been recently discovered as the third gene involved in the autosomal dominant form of hypercholesterolemia (Varret et al., 1999; Abifadel et al., 2003). The human PCSK9 is mostly expressed in liver and encodes a 692-residue glycoprotein belonging to the family of protein convertases (Seidah et al., 2003). PCSK9 plays a crucial role in LDLR degradation, where its effect on cholesterol metabolism is mainly performed by changing LDLR recycling process. Circulating PCSK9 binds to LDLR on liver cell surface and leads to LDLR internalization and degradation within the cell (Maxwell and Breslow, 2004). Gain-of-function mutations in *PCSK9* lead to an elevation in serum level of PCSK9, LDLR degradation, an increase in serum level of cholesterol, and consequently a higher risk of hypercholesterolemia (Abifadel et al., 2003). Hence, PCSK9 has been considered as a new target for hypercholesterolemia treatment. Most current strategies are based on PCSK9 protein level reduction using monoclonal antibodies and gene silencing methods (Sinning and Landmesser, 2017; Wong et al., 2017).

microRNAs (miRNA, miR) are a group of small, endogenously made non-coding RNAs regulating a wide-range of molecular pathways including cell proliferation, apoptosis, and differentiation (Bartel, 2004; Jovanovic and Hengartner, 2006; Lin and Gregory, 2015). A role of miRNAs in lipid and cholesterol metabolism has been discussed in various studies. miR-122 and miR-33 have been shown to play an important role in fatty acid metabolism. Inhibition of miR-122, a liver-specific microRNA, in mice resulted in decreased level of plasma cholesterol and increased hepatic fatty acid oxidation. Cholesterol synthesis rate was also decreased upon miR-122 inhibition (Esau et al., 2006). miR-33 is one of the well-studied miRNAs related to lipid and cholesterol metabolism. miR-33 is encoded within the sterol-regulatory element-binding factor-2 (SREBP2), a transcription factor that regulates lipid metabolism and reduces cellular cholesterol export via inhibition of translation of the cholesterol export pump ABCA1 (Gerin et al., 2010; Rayner et al., 2010). Moreover, Davalos et al. (2011) reported that miR-33 also regulates insulin metabolism in the liver via targeting insulin receptor substrate 2, an essential component of the insulin-signaling pathway in the liver (Davalos et al., 2011). In 2015, Alvarez et al. introduced miR-27 as a regulator of cholesterol metabolism that can directly interact with the LDLR transcript and have an effect on PCSK9 level indirectly through an unknown pathway (Alvarez et al., 2015).

In this study, we predicted miRNAs targeting the 3′-UTR of PCSK9 and then experimentally validated their effects on PCSK9 expression level. Our data revealed that miR-191, miR-222, and miR-224 can bind the 3′ end of the PCSK9 mRNA and thus regulate PCSK9 expression. Our data suggest that miR-191, miR-222, and miR-224 could be considered as a new molecular targets for manipulating PCSK9 expression and CVD therapies.

## Materials and methods

### Predicting PCSK9-targeting miRNAs using bioinformatics tools

To predict miRNAs capable of targeting PCSK9 the following bioinformatics tools were employed: TargetScan (http://www.targetscan.org/vert_71/), DIANA tools (http://diana.imis.athena-innovation.gr/DianaTools/index.php), miRDB (http://mirdb.org/), miRmap (http://mirmap.ezlab.org/), miRTarBase (http://mirtarbase.mbc.nctu.edu.tw/), and miRanda (http://www.microrna.org/microrna/home.do).

### Cell culture

Huh7 and A549 cell lines were cultured in DMEM media (Invitrogen, USA), supplemented with 100 U/ml penicillin, 100 μg/ml streptomycin (Sigma, USA), as well as 10% fetal bovine serum (FBS) (Invitrogen, USA), and incubated in 37°C with 5% CO2. HEK293T and HepG2 cells were cultured in DMEM-F12 (Invitrogen, USA) containing 10% FBS, 100 U/ml penicillin and 100 μg/ml streptomycin.

### Expression quantification of selected miRNAs and PCSK9 in cell lines

Cultured cells were lysed using RiboEx (Genall, South Korea) and RNA was extracted guanidinium-thiocyanate phenol-chloroform separation protocol, according to the manufacturer's instructions (Chomczynski and Sacchi, 1987). To eliminate any possible DNA contamination, RNA was treated with DNase I enzyme (Fermentas, USA). One unit of DNase I enzyme and 1 μl of buffer were added to 1 μg of the extracted RNA and incubated for 30 min at 37°C. Enzyme's inactivation was performed by adding 1 μl of 50 mM EDTA and incubation at 65°C for 10 min. cDNA was synthesized using PrimeScript first strand cDNA synthesis kit (TAKARA, Japan), according to the manufacturer's protocol. In brief, 0.5 μl of RT enzyme, 2 μl of RT buffer and 1 μl of random hexamer were mixed with 5 μl of DNase-treated RNA and incubated for 15 min at 37°C, and 5 s at 85°C for enzyme inactivation. For evaluating miRNAs expression, 1 μg of total RNA was polyadenylated using polyA polymerase (NEB), according to manufacturer protocol, and reverse transcribed into cDNA using anchored-oligo dT primers (Supplementary Table 1). The expression levels of *PCSK9* and the nominated miRNAs were determined using specific primers listed in Supplementary Table 1. Real-time PCR was performed using BIOFACT™ 2X real-time PCR master mix (for SYBR Green I; BIOFACT, South Korea).

### miRNAs gene cloning and overexpression

The predicted miRNA genes were PCR amplified and cloned into pEGFP-C1 vector (Clontech, Japan) downstream of the GFP gene. To examine the effects of selected miRNAs on *PCSK9* expression, the miRNAs were overexpressed in the HepG2 cell line and the mRNA expression level of PCSK9 was measured after 48 h post-transfection. Then, the transfected cells were lysed by RiboEx reagent and PCSK9 mRNA level was determined by real-time PCR, as mentioned before.

### Cloning of PCSK9's 3′-UTR and 3′-UTR mutated form

To clone the 3′-UTR of PCSK9, we used archived frozen human genomic DNA that had been extracted from a blood sample of a male volunteer. Informed consent had been obtained from the participant for the use of his blood sample. This study had been approved by the Clinical Research Ethics Committee of Shariati Hospital, Tehran University of Medical Sciences. For PCSK9 3′-UTR cloning, the region corresponding to the 3′-UTR and its antisense, used as the scrambled control, about 1,300 nt fragment of the human PCSK9 was PCR amplified using specific primers and cloned into psiCHECK-2 vector (promega, USA) downstream of the Renilla gene both in sense and antisense directions. As control, three different mutant constructs in which the whole binding site of nominated microRNAs was eliminated, were built using SOEing PCR (for primers sequences please see Supplementary Table 1). In miR-224 mutant construct both putative microRNAs target sites were deleted from 3′-UTR sequence.

### HEK293T transfection

Three hundred nanograms of miRNA-expressing vectors and 150 ng wild type or mutated 3′-UTR constructs were co-transfected in HEK239T (cultured in 48-well plates) using lipofectamine 2000 (Invitrogen, USA). Psicheck-2 and pEGFP-C1 mock vectors were also transfected as controls for luciferase assay and transfection, respectively. Transfections were performed in triplicate, and its efficiency was monitored by GFluorescent microscopy (Nikon TE2000S, Japan) after 36 h.

### Luciferase assay

Forty-eight hours after HEK293T transfection, luciferase reporter assay was performed using Dual-Luciferase Reporter Assay System (Promega, USA) by luminometer (Titertek-Berthold, Germany) according to the manufacturer's protocol. Briefly, cell media was removed completely and lysis buffer was added to each well. After 20 min, firefly luciferase activity, as a control, was measured by adding LARII reagent and after that Renilla activity was determined by Stop & Glo reagent.

### Statistical analysis

ΔΔCt method was used for real-time RT-PCR data analysis and gene expression determination. GraphPad Prism 6 was also used for real-time RT-PCR, luciferase assay data analysis and *p*-value calculation. *P* < 0.05 was considered as statistically significant for all experiments.

## Results

### Bioinformatics prediction of PCSK9-targetting miRNAs

**Figure 1:**
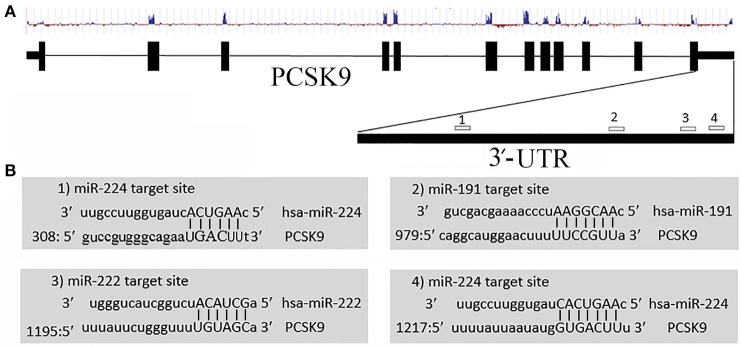
Predicted miR-191, miR-222, and miR-224 target sites on PCSK9 transcript. **(A)** Exon, intron, conservation status, and miRNAs target sites on PCSK9 sequence. We only selected miRNAs targeting the 3′-UTR. **(B)** MiRNAs target sites and their nucleotide hybridization status.

We employed various target prediction programs to predict miRNAs targeting 3′-UTR of the PCSK9 transcript. These programs predicted several miRNAs, and according to common predicted data miR-191, miR-222, and miR-224 were selected for further investigation. miR-191 and miR-222 have one target site, whereas miR-224 possess two target sites on PCSK9 3′-UTR (Figure 1).

### A reciprocal pattern of expression between predicted miRNAs and PCSK9 in studied cell lines

**Figure 2:**
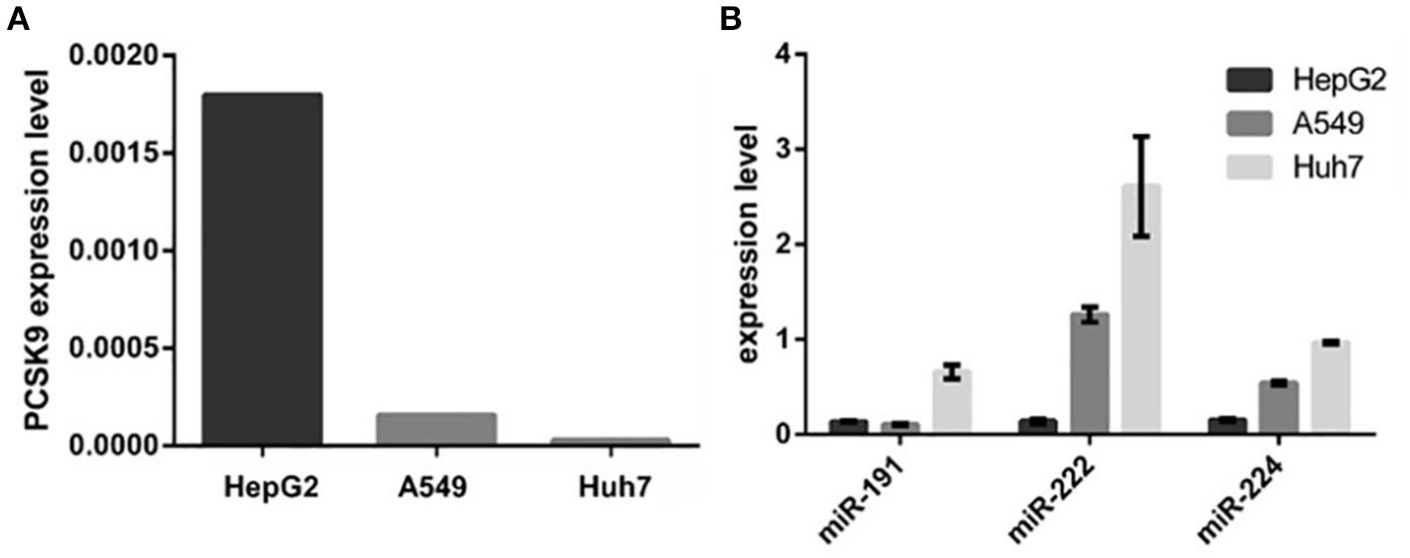
PCSK9 and the selected miRNAs relative expressions in HepG2, A549, and Huh7 cell lines. **(A)** PCSK9 expression in three cell lines. PCSK9 is expressed in HepG2, Hepatic cell line, more than A549 and Huh7 cell lines. GAPDH expression was used as an internal control. **(B)** miR-191, miR-222, and miR-224 expression levels in three cell lines. In contrast to PCSK9, all three miRNAs are expressed in Huh7 cells more than two other cell lines. MiRNAs expression level were normalized to u48 expression, as an internal control.

We examined miR-191, miR-222, miR-224, and *PCSK9* expression levels in three different cell lines including HepG2, Huh7, and A549. Results revealed that *PCSK9* expression showed the highest level in HepG2 cells, and the lowest level in Huh7 cells. In contrast, HepG2 cells showed the lowest expression levels and Huh7 cells showed the highest expression levels for miR-191, miR-222, and miR-224 (Figure 2).

### miR-191, miR-222, and miR-224 overexpression diminished PCSK9 mRNA level in HepG2 cell line

**Figure 3:**
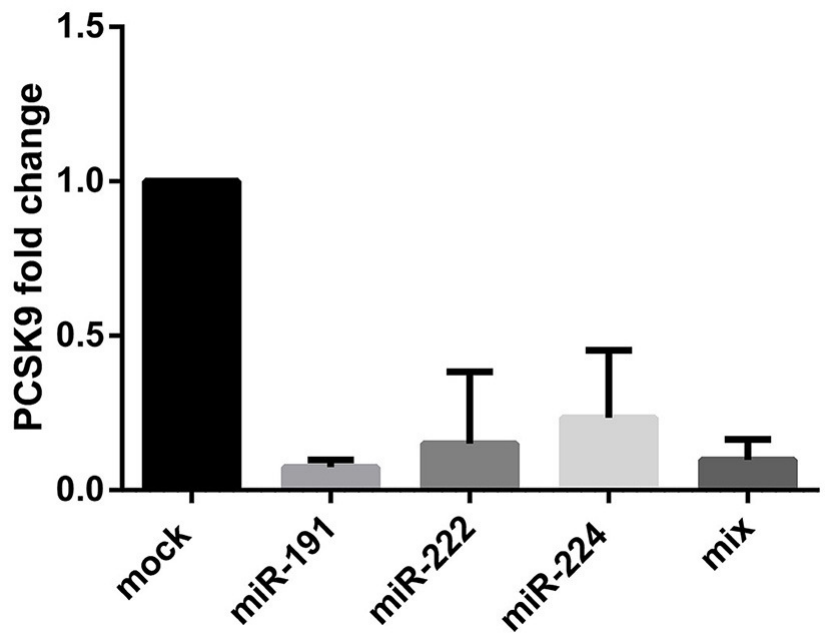
PCSK9 mRNA fold change after miR-191, miR-222, miR-224, and the mixture of all three miRNAs overexpression in HepG2 cell line. PCSK9 mRNA level declined after the miRNAs overexpression in all four conditions. GAPDH gene was used as an internal control for PCSK9 expression normalization.

Expression vectors containing the precursors of miR-191, miR-222, and miR-224 were transfected into HepG2 cell line and PCSK9 mRNA level was measured 48 h post-transfection. Our data demonstrated a significant downregulation of PCSK9 in the cells transfected with vectors overexpressing miR-191, miR-222, and miR-224 (Figure 3), compared to the cells transfected with mock vector. Similar results were obtained for the cells transfected with a mixture of all three miRNAs.

### Direct interaction of miR-191, miR-222, and miR-224 with the 3′-UTR of PCSK9

**Figure 4:**
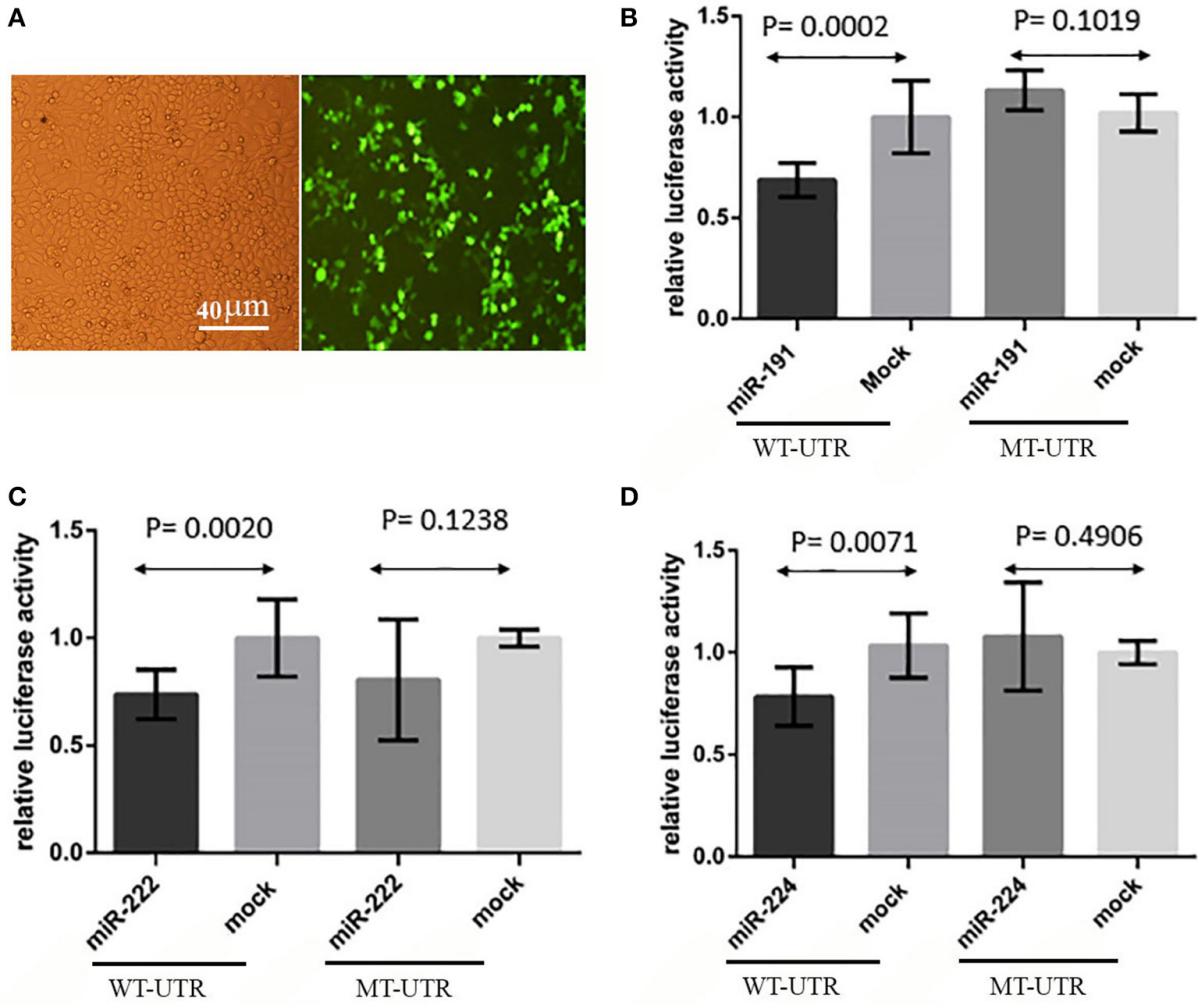
Luciferase assay results. **(A)** HEK293T transfection, GFP signal showed that transfection rate was about 70% and pEGFP-C1 vectors were functional. **(B)** Relative luciferase activity was decreased in wild type 3′-UTR (WT-UTR) (*P* = 0.0002) after miR-191 overexpression, but there was no significant alteration in relative luciferase activity when miR-191 target site was deleted from 3′-UTR (MT-UTR) (*P* = 0.1019). **(C)** miR-222 significantly decreased relative luciferase activity in wild type 3′-UTR (WT-UTR) condition (*P* = 0.0020), but there was no significant change in relative luciferase activity when miR-222 target site was omitted from the 3′-UTR (MT-UTR) (*P* = 0.1238). **(D)** miR-224 overexpression effect on relative luciferase activity in wild type 3′-UTR (WT-UTR) and mutated 3′-UTR (MT-UTR) conditions. miR-224 significantly decreased relative luciferase activity in WT-UTR (*P* = 0.0071), but there was no significant change in relative luciferase activity when miR-224 target site was deleted (MT-UTR) (*P* = 0.4906).

A direct interaction of the predicted miRNAs with the 3′-UTR of PCSK9 was examined by means of luciferase assay. The obtained data demonstrated that miR-191 overexpression significantly decreased the relative luciferase activity (*P* = 0.0002) in the cells co-transfected with a vector containing a luciferase-coding sequence upstream of the wild type 3′-UTR of PCSK9. In contrast, a mutated form of PCSK9 3′-UTR containing a deletion for miR-191 target site failed to do the same effect on transfected cells (*p* = 0.1019). miR-222 (*P* = 0.0020) and miR-224 (*P* = 0.0071) showed similar effects on diminishing the relative luciferase activity in the transfected cells (Figure 4). Similar to what we observed for miR-191, miR-222 (*P* = 0.1238) and miR-224 (*P* = 0.4906) had no significant effects on luciferase activity when their putative target sites were omitted from the 3′-UTR.

## Discussion

Familial hypercholesterolemia is the major cause of cardiovascular disease and the most common inherited form of dyslipidemia. Heterozygous mutations in LDL Receptor (Davis et al., 1986), Apolipoprotein B (Innerarity et al., 1987), PCSK9 (Abifadel et al., 2003), and Apolipoprotein E (Marduel et al., 2013) are linked to hypercholesterolemia. Secreted PCSK9 binds to the LDLR on the cell surface and promotes its degradation in lysosomes thereby inhibiting the LDLR recycling pathway. This process leads to LDL-cholesterol accumulation in circulation (Maxwell et al., 2005; Nassoury et al., 2007). FH management is mainly based on reducing plasma LDL-Cholesterol, and based on this logic a PCSK9 inhibitor can be used as a new therapy for hypercholesterolemia.

Recently, miRNAs have been introduced as important regulators of gene expression, and their expression alteration have been reported in different disease conditions including CVD. These small (18–23 nucleotides) non-coding RNAs control a variety of cellular processes ranging from cell cycle to lipid metabolism. Here, by using bioinformatics and *in vitro* functional analysis, we introduced miR-191, miR-222, and miR-224 as natural regulators of PCSK9. Moreover, we confirmed that the three miRNAs directly target PCSK9 and regulate its expression.

miRNAs regulate gene expression in different ways including transcription regulation, mRNA degradation, translation inhibition or even translation activation (Vasudevan et al., 2007; Bartel, 2009). Here, our data revealed that upon miRNAs overexpression in HepG2 cell line, PCSK9 mRNA level decreased significantly. This finding means that these miRNAs are capable of regulating PCSK9 expression, post-transcriptionally.

There are some studies linking miR-191, miR-222, and miR-224 to carcinogenesis including hepatocellular carcinoma. Interestingly, the aforementioned miRNAs could act as an oncogene or tumor suppressor (Nagpal and Kulshreshtha, 2014; Zhang et al., 2015; Okajima et al., 2016). miR-191 is expressed as part of miR191/425 cluster, which is highly conserved, and is found to be deregulated in different disease conditions such as type 2 diabetes (Zampetaki et al., 2010; Nagpal and Kulshreshtha, 2014). In hepatic cellular carcinoma, hypomethylation of mir-191 locus causes its elevated expression and promotes the epithelial-to-mesenchymal transition (He et al., 2011). miR-191 targets EGR1 (Early growth response protein 1), a zinc finger transcription factor which has binding site on PCSK9 promoter and could regulate its expression in different physiological status (Di Leva et al., 2013). Noting this relation, it might be safe to say that miR-191 could regulate PCSK9 in both direct and indirect ways.

miR-222 has been introduced as a promising clinical biomarker for metabolic diseases. Vickers et al. reported that miR-222 level in circulating HDL was 8.2-fold higher in familial hypercholesterolemia patients than HDL from normal subjects (Vickers et al., 2011). miR-222 also regulates insulin sensitivity in adipocyte and it was found to be significantly higher in plasma of obese human patients (Ortega et al., 2013). Additionally, miR-222 has been reported to play important roles in many physiological and pathological processes in the cardiovascular system, where its deregulation has been implicated in many cardiovascular diseases. It also plays a role in atherosclerosis and plaque formation (Bazan et al., 2015). Our findings on the ability of miR-222 for regulating the expression of PCSK9, a LDLR negative regulator, is in line with its formerly identified roles in lipid metabolic diseases and susceptibility for developing obesity and heart failure. Finally, it has been very recently reported that miR-222 and mir-221 inhibit HIV-1 Entry in Macrophages by Targeting the CD4 Viral Receptor (Lodge et al., 2017). Thus, it is possible that miR-221 may also inhibit PCSK9 expression, as the levels of both miR221 and 222 are enhanced by tumor necrosis factor alpha (TNF-α).

The role of miR-224 in lipid metabolism has been investigated in several studies, and as our data also demonstrated it could be considered as a major regulator in lipid metabolic pathways. Peng et al. reported that miR-224 is a negative regulator of adipocyte differentiation. This miRNA also regulate fatty acid metabolism by directly targeting Acyl-CoA synthetase long-chain family member 4 (ACSL4), which is an essential enzyme in fatty acid metabolism (Peng et al., 2013). Our data revealed that miR-224 could negatively regulate PCSK9 expression level by directly targeting its transcript. Our findings are in accordance with former studies that introduced miR-224 as a regulator in lipid metabolism and of PCSK9 in particular (Bai et al. 2017).

Overall, our findings demonstrated that miR-191, miR-222, and miR-224 could play important roles in lipid and cholesterol metabolism and participate in developing disease conditions such as hypercholesterolemia and CVD, by targeting PCSK9 which has a critical role in LDLR degradation and cellular LDL uptake. Due to the importance of LDLR and PCSK9 regulation, these miRNAs could be considered as novel biomarkers and therapeutic targets in hypercholesterolemia, obesity and cardiovascular diseases. However, metabolic disorders are multifactorial and caused by complex deregulation in biochemical and cellular signaling pathways. Therefore, further investigations are needed to clarify the possible therapeutic effects of miR-191, miR-222, and miR-224 manipulation on pathobiological pathways.

## Author contributions

PN: experiment design, lab work, data production, data interpretation, writing first draft of MS; FM: lab work, data production, data interpretation, MS edit; MM: experimental design, data interpretation, MS edit; NS: experiment design, data interpretation, MS edit; NGS: experiment design, data interpretation, MS edit. SJM: project design, data interpretation, manuscript final edit.

### Conflict of interest statement

The authors declare that the research was conducted in the absence of any commercial or financial relationships that could be construed as a potential conflict of interest.

## Supplementary material

Table S1 The sequences of primers designed for SOEing PCR or amplification or PCSK9 and microRNAs used in this study.
