# Supplementary material for: Post-transcriptional Regulation of PCSK9 by miR-191, miR-222, and miR-224
Source: Front Genet. 2017 Nov 27;8:189. doi: 10.3389/fgene.2017.00189 (PMC5711823; doi:10.3389/fgene.2017.00189)
Supplement: Table S1 — The sequences of primers designed for SOEing PCR or amplification or PCSK9 and microRNAs used in this study. [file Table1.DOCX]

Supplementary Table 1

| PCR primers | Gene name | Forward primer | Reverse primer |
| --- | --- | --- | --- |
|  | PCSK9 | AGGGGAGGACATCATTGGTG | CAGGTTGGGGGTCAGTACC |
|  | miR-191 | CAACGGAATCCCAAAAGCAG | CCAGTGAGCAGAGTGACG |
|  | miR-222 | AGCTACATCTGGCTACTGG | CCAGTGAGCAGAGTGACG |
|  | miR-224 | CAAGTCACTAGTGGTTCCGT | CCAGTGAGCAGAGTGACG |
|  | anchored-oligo dT | CCAGTGAGCAGAGTGACGAGGACTCGAGCTCAAGCTTTTTTTTTTTTTTTTTN(C,G,A,T) | |
| SOEing PCR primers* | Construct name | Forward primer | Reverse primer |
|  | miR-191 mutant | F1: CCGTTGCCATCTGCTGCC | R1:AGTGAATCAGGCCTGGGTGACAGGCATCGTTCTGCCATCA |
|  |  | F2:TGATGGCAGAACGATGCCTGTCACCCAGGCCTGATTCACT | R2:TCAAGTCTATGCAAGAGTTAGGACA |
|  | miR-222 mutant | F1: CCGTTGCCATCTGCTGCC | R1:TTGCGGCCGCTATCTTCAAGTTACAAAAGCAAAACAGG |
|  | miR-224 mutant | F1: CCGTTGCCATCTGCTGCC | R1:GCACGGAACAAGAGCTCAATAATCGGCACATTGGGAGCAG |
|  |  | F2:CTGCTCCCAATGTGCCGATTATTGAGCTCTTGTTCCGTGC | R2:TCAAGTCTATGCAAGAGTTAGGACA |

*These primers were used for microRNAs target site deletion from 3ʹ-UTR. miR-224 had two target sites and both sites were eliminated using SOEing PCR.
